# Dopamine modulation of learning and memory in the prefrontal cortex: insights from studies in primates, rodents, and birds

**DOI:** 10.3389/fncir.2014.00093

**Published:** 2014-08-05

**Authors:** M. Victoria Puig, Jonas Rose, Robert Schmidt, Nadja Freund

**Affiliations:** ^1^The Picower Institute for Learning and Memory, Department of Brain and Cognitive Sciences, Massachusetts Institute of TechnologyCambridge, MA, USA; ^2^Animal Physiology, Institute of Neurobiology, University of TübingenTübingen, Germany; ^3^BrainLinks-BrainTools, Department of Biology, Bernstein Center Freiburg, University of FreiburgFreiburg, Germany; ^4^Department of Psychiatry and Psychotherapy, University of TübingenTübingen, Germany

**Keywords:** prefrontal cortex, learning and memory, dopamine receptors, executive function, working memory, neuromodulation, evolution

## Abstract

In this review, we provide a brief overview over the current knowledge about the role of dopamine transmission in the prefrontal cortex during learning and memory. We discuss work in humans, monkeys, rats, and birds in order to provide a basis for comparison across species that might help identify crucial features and constraints of the dopaminergic system in executive function. Computational models of dopamine function are introduced to provide a framework for such a comparison. We also provide a brief evolutionary perspective showing that the dopaminergic system is highly preserved across mammals. Even birds, following a largely independent evolution of higher cognitive abilities, have evolved a comparable dopaminergic system. Finally, we discuss the unique advantages and challenges of using different animal models for advancing our understanding of dopamine function in the healthy and diseased brain.

## INTRODUCTION

A major function of executive control is the flexible adaptation to our ever-changing environment. The executive circuits of the brain must, therefore, not only monitor and maintain current behavioral goals but also incorporate new goals and rules. This updating can come in the form of a quick integration of previously acquired knowledge when, for example, a well-known stimulus informs an animal of a change in reward contingencies. In many cases, however, such updating requires new learning, for example when a new stimulus is encountered for the first time. Executive functions are commonly ascribed to the prefrontal cortex (PFC) and frontostriatal networks. The function of these circuits relies heavily on neuromodulation, in particular on dopamine (DA). The aim of this review is to outline the contribution of DA and its receptors in the PFC to learning and memory processes across different species.

We will first introduce studies in the mammalian brain in the sections on humans, non-human primates, and rodents. Due to the challenges of investigating the role of DA transmission in human PFC, we focus the human section on studies utilizing systemic injections of DA agents and impairments of DA transmission in patients with a variety of neurological and psychiatric disorders. The non-human primate and rodent sections review behavioral studies conducted during local manipulations of the DA system in the PFC. While the dopaminergic system in different mammalian species follows largely the same organization, some conceptual and terminological differences can make a comparison of data across species difficult (**Box 1**). For a comparative perspective, we will then outline behavioral studies conducted in birds where local manipulations of the DA system were implemented in a structure equivalent to the mammal PFC, the nidopallium caudolaterale (NCL; Jarvis et al., 2005). Such a comparison is of particular interest given the large evolutionary gap between these species. The lines of birds and mammals separated around 300 million years ago, long before many of the cognitive functions attributed to the PFC evolved (Jarvis et al., 2005; Reiner et al., 2005; Jarvis, 2009; Rose et al., 2009a). In spite of this distance, birds and mammals (with the exception of humans and apes) are largely on par when it comes to cognitive abilities (Emery and Clayton, 2004; Kirsch et al., 2008, 2009). This implies a parallel or convergent evolution of cognition between the species (Emery and Clayton, 2004; Güntürkün, 2012). As a result of this independent evolution, we see stark differences in brain organization between birds and mammals (Jarvis et al., 2005). Most notably, the avian telencephalon does not show the laminar organization of the mammalian cortex. However, other organizational principles were preserved or evolved independently in both lines. This can be taken as a hint of narrow neurobiological constraints in the evolution of a given cognitive ability (Colombo and Broadbent, 2000; Güntürkün, 2005a).

**Box 1. Conceptual/terminological differences between species.**

When comparing the function of prefrontal DA across species it is important to clarify the terminology used in the different fields of research. As reviewed here, prefrontal DA plays an important role in learning and memory and an extensive body of literature is concerned with its role particularly in working memory (WM). In general, the term WM is strongly associated with its original definition by Baddeley and Hitch (1974), who famously proposed that systems for sensory storage (phonological loop, visuospatial sketchpad, and more recently, an episodic buffer) are governed by a central executive (Baddeley, 1992, 2000). The gist of this definition is that an interconnected neural system allows the brief storage of information and, importantly, its manipulation.

In primates, a seminal contribution to the understanding of this system was the discovery of Fuster and Alexander (1971) of “delay cells” in the PFC. These neurons show increased activity during the delay period of WM tasks maintaining the memory of a stimulus. Consequently, in primates including humans, WM is often modeled as “active memory” (Zipser et al., 1993; Durstewitz, 2009), a system that holds information in memory by sustaining neural activity for a few critical seconds.

Research in rodents commonly uses a broader definition of WM, that refers to *“a collection of processes that include the temporary storage of information, as well as executive functions that mediate the manipulation and retrieval of trial-unique information to guide action after both short (seconds) and longer (minutes to hours) delays*” (Phillips et al., 2004; see also: Mizumori et al., 1987; Floresco and Phillips, 2001). Importantly, this definition includes a much larger range of delays (seconds to many hours) compared to what is typically used in humans and non-human primates (seconds). Consequently, in rodents, the definition of WM does not necessarily refer to active memory maintenance by delay cells but might rely on different mechanisms that could be classified as learning mechanisms in primates. Thus, it is important to pay attention to the specific paradigms and definitions used when comparing results across species.

The definition of WM typically used in avian research was developed in parallel to the definition in humans (Honig, 1978). Both concepts are largely comparable with the exception that no phonological loop is conceptualized in birds. The delay durations in avian research are largely comparable to those in the primate literature and active information maintenance by delay activity is generally assumed to be the key mechanism of WM (Miller et al., 1996; Güntürkün, 2005a).

Taken together, there are fundamental terminological differences between species and it is important to keep these in mind when comparing results across species. In particular, the vast differences in delay duration used in different paradigms could potentially engage distinct neural mechanisms – what is called WM in one species might be viewed as a learning mechanism in another.

## ANATOMY OF THE DOPAMINE SYSTEM IN THE PREFRONTAL CORTEX

**FIGURE 1:**
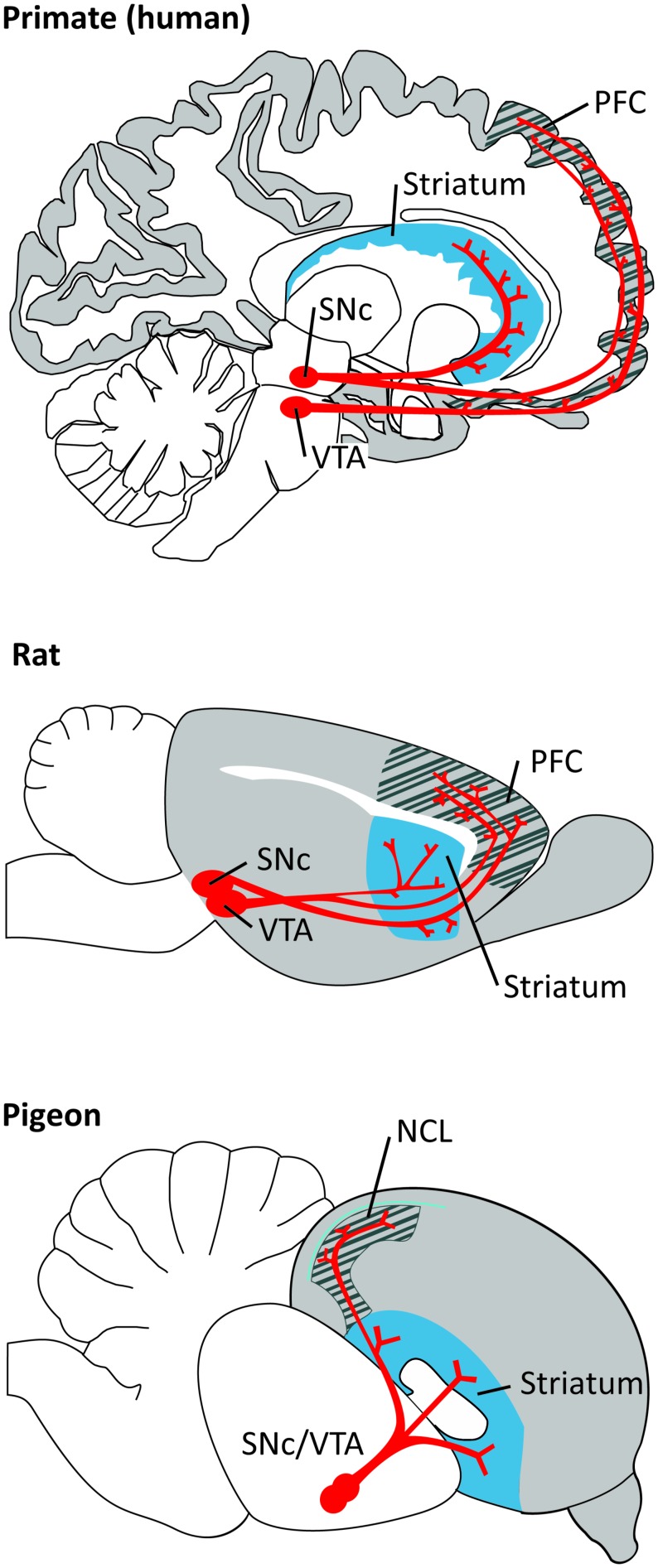
**Dopaminergic projections (in red) from the ventral tegmental area (VTA) and substantia nigra pars compacta (SNc) to the PFC/NCL and striatum in the brain of a primate (human), a rat, and a pigeon**. Pallial (cortical) areas across species are shaded in gray, the hatched area denotes the PFC/NCL, striatal areas are shaded in blue. Note that, in all species, DA neurons in both dopaminergic nuclei project to several subregions of the PFC/NCL and striatum.

The anatomy of the dopaminergic system is very similar between all mammals and birds (for extensive review, see Durstewitz et al., 1998, 1999b; Björklund and Dunnett, 2007). DA neurons can be identified by the expression of several catecholamine-synthesizing enzymes, tyrosine hydroxylase (TH), aromatic amino acid decarboxylase (AADC), and dopamine-b-hydroxylase (DBH). With modern immunohistochemical techniques it has been possible to map out in detail the location of DA neurons and their specific projections. DA neurons originate in several neighboring midbrain nuclei, being the substantia nigra pars compacta (SNc; A9) and the ventral tegmental area (VTA; A10) the ones projecting to the forebrain. The total number of TH-positive cells in VTA and SNc (bilateral count) is ∼20.000–30.000 in mice and ∼40.000–45.000 in rats. This number increases considerably in primates, 160.000–320.000 in monkeys and 400.000–600.000 in young humans. DA neurons send afferents to many target areas, including the several regions of the frontal cortex, with the striatum being the most densely innervated target (Björklund and Dunnett, 2007; **Figure 1**). PFC-projecting DA neurons are intermingled in VTA and SNc both in primates and in rodents. However, the PFC in primates is much more extensively innervated by midbrain DA afferents than in rodents (Thierry et al., 1973; Lindvall et al., 1978; Swanson, 1982; Descarries et al., 1987; Lewis and Sesack, 1997; Björklund and Dunnett, 2007).

Postsynaptically, DA exerts its actions within the PFC/NCL via receptors grouped in two major families, D1-like receptors (D1 and D5 in mammals; D1A and D1B in birds) and D2-like receptors (D2, D3, and D4 in mammals and birds), but D1-like receptors are expressed to a greater extent than D2-like receptors (Lidow et al., 1991; Durstewitz et al., 1998; Seamans and Yang, 2004; de Almeida et al., 2008; Santana et al., 2009; de Almeida and Mengod, 2010). In birds, the D1-like family is extended to include an additional receptor (D1D; Callier et al., 2003; Kubikova et al., 2010). Both families are G-protein-coupled receptors that exert slow changes of activity in the cells and act as functional neuromodulators. D1-like receptors show low affinity for DA, whereas D2-like receptors show higher affinity (Seamans and Yang, 2004). For the sake of clarity, we will abbreviate D1-like and D2-like receptors as D1R and D2R, respectively, and will point to a specific receptor subtype whenever necessary.

Interestingly, dopaminergic signaling in the PFC depends on brain maturation and the PFC is the brain structure that matures last (Gogtay and Thompson, 2010). Analyses of human postmortem brain tissue reveal that the levels of mRNA expression of the D2R and D5R subtypes in PFC are highest in neonates and infants and decrease with age, whereas the D1R subtype mRNA expression and protein levels increase with age and are highest in adulthood (Rothmond et al., 2012). By contrast, both in rats and non-human primates, densities of the D1R and D2R subtypes peak during adolescence and decrease in adulthood (Rosenberg and Lewis, 1994; Andersen et al., 2000). In songbirds, D1R and D2R subtypes in the song nuclei increase with age and peak during adolescence (Kubikova et al., 2010). The developmental patterns of related brain regions in non-songbirds are still unclear.

## NEUROPHYSIOLOGY OF DA NEURONS

“Classic” DA neurons show phasic activations (short duration bursts of action potentials) following unpredicted reward coding a quantitative “prediction error” signal, namely the difference between received and predicted reward value. A reward that is better than predicted elicits an activation (positive prediction error response), a fully predicted reward draws no response, and a reward that is worse than predicted induces a decrease in activity (negative error response; Schultz et al., 1993; Schultz, 2007, 2013). These prediction error responses of DA cells have been closely related to reinforcement learning models which assign a functional role of DA in modulating cortico-striatal inputs through a reward-prediction error teaching signal (Schultz, 1997, 2002; Morris et al., 2004, 2006; Pan et al., 2005, 2008). In fact, fast DA release consistent with these reward predicting signals of DA neurons has been measured in nucleus accumbens during associative learning (Phillips et al., 2003; Day et al., 2007). Besides “classic” reward-prediction error responses, phasic DA cell firing patterns also include responses to salient and aversive sensory stimuli (Horvitz, 2000; Joshua et al., 2008; Brischoux et al., 2009; Matsumoto and Hikosaka, 2009).

Dopamine neurons also exhibit tonic firing driven by pacemaker-like membrane currents (Grace and Bunney, 1984; Grace, 1991; Goto et al., 2007). The functional relevance of this tonic DA release is unknown. Transient suppression of tonic spiking in DA neurons follows the omission of expected reward, somehow implicating this spiking pattern in reward-based learning (Tobler et al., 2003). Recent work has shown that DA release in the striatum increases gradually (ramps up) as rats expect distant reward, perhaps providing motivational drive (Howe et al., 2013). However, these types of signals have not been described in PFC.

Which of these DA signals reaches the PFC remains currently unclear. While phasic DA prediction error signals could be used as a signal to transiently boost working memory (WM) of the corresponding stimuli (Cohen et al., 2002; O’Reilly et al., 2002), it has also been argued that mostly slower, tonic DA signals are relevant in PFC. Moreover, the phasic components of DA cell firing might be transmitted via co-release of glutamate (Seamans and Yang, 2004; Lavin et al., 2005; Castner and Williams, 2007; Sheynikhovich et al., 2013). For computational models of DA function in PFC this has two main consequences. Firstly, the timescales of tonic DA would constrain functional roles to rather general cognitive states such as arousal or attention. Secondly, DA function in PFC circuits should be carefully contrasted with known features of the putatively fast, phasic, signals of the nigrostriatal system.

In general, heterogeneity among DA cells points to additional functional aspects that are not covered by classic reinforcement learning descriptions (Berridge, 2007; Redgrave et al., 2008; Bromberg-Martin et al., 2010; Morris et al., 2010). While functional roles of VTA and SNc neurons share common properties (Ilango et al., 2014), overall evidence for different functional groups among DA cells has been emerging (Brischoux et al., 2009; Matsumoto and Hikosaka, 2009; Lammel et al., 2012; Watabe-Uchida et al., 2012). Moreover, the heterogeneity in DA cell activity patterns is probably related to heterogeneity in the anatomical pathways; DA neurons contribute to reward or aversion depending on whether they are activated from the laterodorsal tegmentum or the lateral habenula, respectively (Lammel et al., 2012). For these reasons, it has been difficult to dissociate the behavioral correlates of DA release between the projection pathways to the striatum and PFC.

## HUMAN STUDIES

Investigating the direct role of DA signaling in human PFC during learning and memory brings quite a few challenges and, consequently, only few studies address this question. DA receptor agonists and antagonists cannot be injected locally, restricted to the PFC, and have to be administered systemically in humans. Our knowledge about the role of DA transmission in the human PFC, therefore, comes from studies combining imaging of the brain with other manipulations such as systemic pharmacology or transcranial magnetic stimulation, genetic profiling, and from work in patients with neurological and psychiatric disorders.

For instance, a recent fMRI study has revealed a connection between context dependent WM and dopaminergic signaling in human PFC (D’Ardenne et al., 2012). The authors first identified by fMRI that the dorsolateral PFC was involved in the encoding of the context. Selective disruption of activity in this region with transcranial magnetic stimulation adversely impacted performance of the participants, causally implicating PFC in context encoding. PFC activity during the task was then found to correlate with phasic responses in the VTA and SNc. Based on these results, the authors suggest that phasic DA signals regulate the encoding and updating of context representations in the PFC.

In the 1970s, it was postulated that hypofrontality (i.e., decreased blood flow in the PFC) underlies mental disorders and impaired cognitive function (Ingvar and Franzén, 1974). In the context of schizophrenia, it was proposed that an excess of DA in the mesolimbic system causes the positive symptoms via hyperstimulation of D2R in the basal ganglia, whereas the cognitive and negative symptoms follow insufficient D1R activation in the frontal cortex (Abi-Dargham and Moore, 2003; Abi-Dargham, 2004). We now know that DA hypofrontality by itself cannot fully explain schizophrenia or other complex mental disorders. Impairments in PFC dopaminergic signaling and genetic profiling in these patients, however, have provided valuable information about the role of PFC DA in learning and memory. For example, schizophrenia patients exhibit imbalances in PFC dopaminergic signaling as determined by imaging approaches (Seeman, 1987; Okubo et al., 1997; Thompson et al., 2014), and show deficits in learning and WM (Kalkstein et al., 2010) that correlate with genetic variations in DA related genes (Glatt et al., 2003; Vereczkei and Mirnics, 2011). In Parkinson’s disease (PD) patients, degeneration of neurons in the SNc results in decreased phasic and tonic PFC DA levels (Scatton et al., 1983; Moustafa and Gluck, 2011), which could explain the cognitive impairments present along with the motor deficits (Narayanan et al., 2013). A more direct involvement of DA in PFC-dependent memory processes was established in PD patients with and without DA medication. In a spatial WM task, subjects had to find tokens in boxes presented on a screen. Subjects that were off the DA precursor levodopa (L-DOPA) made more errors (checking boxes that had already been opened) compared to when they had received L-DOPA, indicating that DA is required for proper spatial WM performance. Surprisingly, visual learning and memory was not affected by L-DOPA in this task (Lange et al., 1992). Similarly, L-DOPA withdrawal did not affect the performance of PD patients in an N-back task, where WM is assessed when subjects are presented with a series of stimuli and have to indicate when a stimulus is the same as the one n steps back (Mattay et al., 2002). However, in PD patients undergoing deep brain stimulation surgery, microstimulation of the SN disrupts reinforcement learning in a two-alternative probability learning task (Ramayya et al., 2014). Furthermore, research conducted in attention deficit hyperactivity disorder (ADHD) patients, who also display learning and memory deficits, have also provided some insight into the role of DA in learning and memory (Brown, 2006; Alderson et al., 2013). In these patients, the size of the PFC is reduced (Seidman et al., 2005), and genes involved in dopaminergic pathways are altered (Gizer et al., 2009). Taken together, the results from work in schizophrenia, PD, and ADHD patients point to an abnormal DA transmission as being responsible for behavioral deficiencies in some learning and memory tasks that depend heavily on PFC function.

Genetic studies have also provided valuable insight into the contribution of the DA system in learning and memory. Individuals with the Val/Val catechol-*O*-methyltransferase (COMT, enzyme that deactivates catecholamines) polymorphism [Val(108/158)Met] exhibit higher COMT activity that correlates with lower DA levels in the PFC (Chen et al., 2004), and have a slightly higher risk of developing schizophrenia (Sagud et al., 2010). Moreover, Val/Val carriers perform worse in the Wisconsin card sorting test (WCST) compared to carriers of the Met allele (Egan et al., 2001; Malhotra et al., 2002). The WCST consists of a battery of cognitive tasks that include WM, sensitivity to reinforcement, and behavioral flexibility. In addition, brain imaging studies indicate that Val/Val carriers need greater PFC activity to perform WM tasks (Egan et al., 2001; de Frias et al., 2010). Stress may be another factor that should be taken into consideration. Healthy human subjects under stress perform poorly in WM tasks (Olver et al., 2014) and exhibit exacerbated levels of PFC DA measured by positron emission tomography (PET; Lataster et al., 2011). In line with this finding, subjects with the above mentioned Val/Val COMT alleles and corresponding reduced levels of PFC DA perform better under stress during WM (Buckert et al., 2012).

Early evidence for the involvement of D1R in WM processes comes from work by Müller et al. (1998) that showed that systemic injections of pergolide, a combined D1R/D2R agonist, but not bromocriptine, a D2R agonist, facilitated WM performance in a delayed matching task with delays of 2–16 s. These results implicated D1R and not D2R in WM modulation. The important role of D1R on WM is also suggested by the correlation between the decrease of D1R binding in the lateral PFC and the decrease in WM performance with age (Bäckman et al., 2011). However, in another study, bromocriptine was shown to improve spatial WM while the D2R antagonist haloperidol (a typical antipsychotic drug) impaired it (Luciana and Collins, 1997). Other experiments, though, did not report a general effect of bromocriptine on spatial memory (Kimberg et al., 1997; Müller et al., 1998) nor binding of the D2R agonist [11C]FLB457 correlated with performance on the WCST (Takahashi et al., 2008).

Positron emission tomography studies in humans with the radioactively marked D1R agonist [11C]SCH23390 have revealed an inverted-U relationship between D1R binding in the PFC and performance on the WCST (Takahashi et al., 2008). An inverted-U relationship means that an optimal level of D1R activation is required for best performance and, thus, levels below and above this optimum impair performance. These experiments were meant to confirm results provided by experimentation in monkeys (see below). Further support for an inverted-U relationship between D1R density and WM comes from patients with schizophrenia. Deficits in WM have been associated with both decreased and increased densities of PFC D1R in these patients (Okubo et al., 1997; Abi-Dargham and Moore, 2003). Taken together, receptor studies in humans point to an important role of PFC D1R in WM with an optimal level of activation needed for best performance. By contrast, the involvement of D2R needs further elucidation.

## NON-HUMAN PRIMATE STUDIES

The use of invasive approaches in monkeys has provided valuable insights into the crucial role of PFC DA and its receptors in several higher-order executive functions. In fact, global 6-hydroxydopamine (6-OHDA) induced depletions of DA in the lateral PFC of monkeys allowed to establish early on the critical role of DA in WM (Brozoski et al., 1979). Later, a series of studies showed that there is an increase of extracellular DA in the PFC during WM tasks (Watanabe et al., 1997) that exerts its actions via local D1R (Sawaguchi and Goldman-Rakic, 1991, 1994; Williams and Goldman-Rakic, 1995; Murphy et al., 1996; Collins et al., 1998; Robbins, 2000; Seamans and Yang, 2004; Castner and Williams, 2007; Arnsten et al., 2010). More specifically, local injections of D1R antagonists, but not D2R antagonists, into the lateral PFC of monkeys caused deficits in oculomotor delayed-response tasks; monkeys were less accurate in making memory-guided saccades to remembered locations on the screen. We note that the WM component of the task in these studies was in the order of 1.5 to 6 s, comparable to the human literature. More recent work has evidenced that an optimal level of D1R tone is required for adequate WM performance, and this may be particularly vulnerable to changes in arousal state such as fatigue or stress (Arnsten et al., 2010; Arnsten, 2011). Thus, either too much (under stress) or too little (during fatigue) D1R stimulation impairs performance following an inverted-U shaped curve (Arnsten et al., 1994, 2010; Cai and Arnsten, 1997; Arnsten and Goldman-Rakic, 1998; Goldman-Rakic et al., 2000; Williams and Castner, 2006; Vijayraghavan et al., 2007; Arnsten, 2012). These reports in monkeys agree well with both the deleterious effects of stress on WM performance and the inverted-U relationship between D1R binding and cognitive capabilities reported in human subjects. This inverted-U modulation of D1R also occurs at the level of single PFC neurons engaged in WM. A D1R agonist modulates persistent activity during memory delays following an inverted-U response, whereby low levels of D1R stimulation enhance spatial tuning whereas high levels reduce it (Vijayraghavan et al., 2007). By contrast, D2R have little effect on delay activity and instead modulate the motor component of the task, suggesting some contribution of PFC D2R to motor control function (Wang et al., 2004). Systemic injections of D1R agonists and antagonists also alter the performance of monkeys during WM tasks, but these studies have been reviewed elsewhere (Castner and Williams, 2007).

One general question is why detrimental effects of the “wrong” DA concentration are present in the system in the first place. In other words, what could be functional reasons for decreasing WM performance? Speculatively, these could occur in situations in which the contribution of PFC to behavior is reduced anyway. For example, in high stress, fight or flight mode, behavioral control could be directed to subcortical areas to emphasize speed (Arnsten, 2012; Avery et al., 2013). Alternatively, the fine-tuning of DA concentration could be used to control the “randomness” of behavior to emphasize exploitation or exploration of certain behaviors (Sutton, 1998; Doya, 2002; Parush et al., 2011; Humphries et al., 2012). Specifically, D1R activation might push the PFC toward an exploitation mode by protecting the WM content against distractors (Durstewitz and Seamans, 2002, 2008). In contrast, based on both computational and experimental approaches, D2R activation has been proposed to support behavioral flexibility (exploration; Floresco and Magyar, 2006; Durstewitz and Seamans, 2008; Puig and Miller, 2014). As in physiological situations selective stimulation of D1R or D2R seems problematic, differences in receptor affinities may produce D2R dominated states (very low and very high DA) and D1R dominated states (intermediate DA). While these properties are also well-suited to support the on- and offset of WM-related persistent activity (**Box 2**), it remains unclear whether the timescales of DA modulation of the PFC firing are fast enough (Cohen et al., 2002; O’Reilly et al., 2002; Seamans and Yang, 2004; Lavin et al., 2005; Sheynikhovich et al., 2013).

**Box 2. Computational perspectives on DA,WM, and PFC persistent activity.**

Models of DA effects in the PFC can be categorized based on their biophysical details of description and their assumed DA release patterns. Furthermore, while the neuropsychological definitions of WM seem not always to be consistent across species (**Box 1**), computational studies often focus on the mechanisms underlying persistent activity during delay periods.

An influential early model of DA action in the PFC (Durstewitz et al., 2000a; see also: Durstewitz et al., 1999a), bridged the gap between DA-induced conductance changes and functional roles. In small networks of multi-compartment models of pyramidal cells and interneurons, increased DA levels changed various intrinsic ionic as well as synaptic conductances. Through a differential effect on cells in high and low activity states, these changes lead to a better separation of the network response to target and distractor patterns. In particular, the network ability to maintain a robust representation of the target pattern for more than one second was improved by increased levels of DA. This feature could be a central function of DA release in PFC, to support persistent activity related to WM.

In a similar approach, increasing the dominance of feedback inhibition in the network resulted in an inverted-U shape function of DA concentration and persistent activity, suggesting a close relation to well-known inverted-U shape relations between DA levels and behavioral performance (Seamans and Yang, 2004). Overall, the ability of DA to enhance persistent activity has been verified on different modeling levels, ranging from detailed Hodgkin-Huxley-like compartmental models (Durstewitz et al., 2000b), over extended integrate-and-fire type descriptions (Brunel and Wang, 2001), to more abstract rate models (Chadderdon and Sporns, 2006). However, it remains unclear which level of model detail is necessary to capture all relevant factors of the extremely complex cellular and synaptic effects of DA in the PFC (Seamans and Yang, 2004). It has been argued that the fundamental underlying principle of changing the signal-to-noise ratio is the strengthening of both excitatory and inhibitory transmission (Cohen et al., 2002); in some cases this is achieved through changes in ionic and synaptic conductance (Durstewitz et al., 2000a), and in others through simple changes in the gain of the neural activation function (Servan-Schreiber et al., 1990). Mechanistically, D1R and D2R have been argued to be essential for changing the dynamics of PFC networks during WM. In the state space of PFC pyramidal and interneuron firing rates, baseline and persistent WM activity form two separate attractors. The level of DA controls the distance between these attractors as well as the structure of the underlying energy landscape, and thereby also the probability of noise to cause a switch between the two regimes (Durstewitz and Seamans, 2002). Still, besides the support of persistent activity, there are other aspects of DA function in PFC that might not be captured by the same principles.

While most previous modeling studies focused on the role of prefrontal DA on WM, a recent study emphasized that DA also affects long-term plasticity in the PFC (Sheynikhovich et al., 2013). Through a multi-compartment model of a PFC neuron (modified from Durstewitz et al., 2000a) they demonstrated that DA can control both the sign and amplitude of long-term plasticity. Potential functional roles of DA-mediated long-term plasticity in PFC could lie in the learning of complex high-dimensional representation of task rules and context (Mante et al., 2013; Rigotti et al., 2013). This would also expand the functional role from WM to a more fundamental role in shaping cognitive processes. The interaction of such structural changes with the other roles of DA in changing PFC activity and oscillatory patterns during WM remains one important direction for future computational approaches.

**FIGURE 2:**
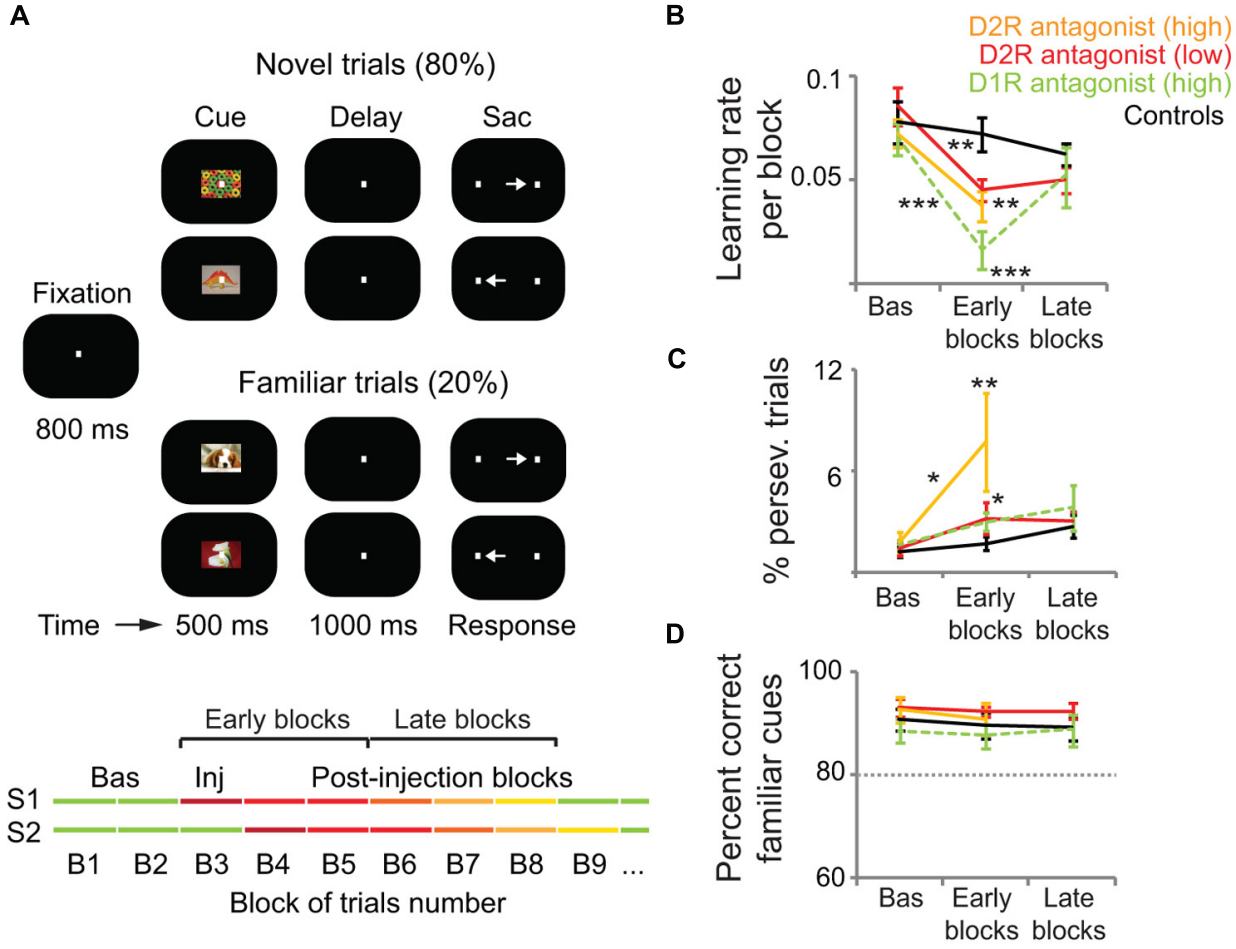
**D1R and D2R in the monkey lateral PFC modulate associative learning but not highly familiar associations**. **(A)** Delayed associative learning and memory task. Animals fixated to start a trial. A cue object was followed by a brief memory delay and presentation of two target dots. Saccade to the target associated with the cue was rewarded with juice drops. Trials were blocked in pairs of novel cues (80% of trials) and pairs of familiar cues (20% of trials). When performance on novel trials reached the learning criteria (80% correct and 30 correct trials per novel cue), novel cues were replaced and a new block of trials started. Monkeys first completed several Baseline blocks (Bas; first green lines). Then, 3 μl of either saline (controls; *n* = 20 sessions), a D1R antagonist (30 μg of SCH23390; *n* = 30 sessions), or a D2R antagonist (high concentration, 30 μg of eticlopride, *n* = 10 sessions; low concentration, 1 μg of eticlopride, *n* = 26 sessions) were pressure-injected in the left lateral PFC (Inj, injection block). Drugs were injected after different numbers of baseline blocks in different sessions (S1–S2) to account for any confounds generated by a systematic behavior of the monkeys. We classified blocks as baseline, “early” (injection block and first two postinjection blocks), or “late” (postinjection blocks 3–5). **(B)** Average learning rates across sessions. We measured the learning rate of each block of trials by fitting a sigmoid distribution to the performance of the monkeys on novel trials using a logistic regression model. Learning rates were the slopes of the fitted distributions. Learning rates decreased significantly after the injection of both D1R and D2R antagonists compared to baseline and post-saline blocks. The D2R antagonist reduced learning rates less than the D1R antagonist. **(C)** Average percent of perseverative errors (consecutive error trials of the same cue). Perseverative errors increased significantly after the injection of both D1R and D2R antagonists compared to baseline and post-saline blocks. The high concentration of the D2R antagonist elicited more perseveration than the other treatments. **(D)** Average percent correct of familiar trials during the baseline, early, and late blocks of trials. Dashed line depicts the 80% threshold used as part of the learning criteria. DA antagonists did not affect the performance of familiar associations. Shown are the mean and SEM. Two-way ANOVA for treatment and blocks as factors. *p < 0.05, **p < 0.01, ***p < 0.001, Tukey’s least significant difference *post hoc* test. Modified from Puig and Miller (2012, 2014).

The monkey lateral PFC has also been implicated in associative stimulus-response learning (Asaad et al., 1998; Pasupathy and Miller, 2005; Histed et al., 2009; Antzoulatos and Miller, 2011; Puig and Miller, 2012, 2014). Reward-prediction error responses of DA cells might be critically involved in these learning processes (Schultz, 1998, 2007, 2013; see above). Consistent with this role in reward prediction, phasic DA release occurs in nucleus accumbens that is dynamically modified by associative learning (Phillips et al., 2003; Day et al., 2007). Thus, it is plausible that these DA signals also play a role in modulating PFC-dependent learning. Indeed, Puig and Miller (2012, 2014) have recently shown that PFC D1R and D2R contribute to stimulus-response learning. Monkeys performed an oculomotor delayed response task where they learned by trial and error associations between visual cues and saccades to a right or left target (**Figure 2A**). Local microinjections of both D1R and D2R antagonists (SCH23390 and eticlopride, respectively) impaired the learning performance of the monkeys, who made more errors and needed more correct trials to learn the associations. The learning impairments correlated with a decrease of neural information about the associations in single prefrontal neurons during both the cue and memory delay (1 s) epochs of the trial. Noteworthy, blocking D1R impaired learning more than blocking D2R, whereas blocking D2R led to more perseverative errors (**Figures 2B,C**). This suggests that PFC D1R contribute to learning more than D2R, whereas the latter are more involved in cognitive flexibility. These complementary roles of D1R and D2R in PFC function agree well with the computational models mentioned earlier that propose that D1R activation helps stabilize new representations once an effective strategy has been identified (exploitation) whereas D2R activation destabilizes PFC network states favoring the exploration of new strategies (i.e., flexible processing; Durstewitz et al., 2000a; Seamans and Yang, 2004; Floresco and Magyar, 2006; Durstewitz and Seamans, 2008).

Contrary to the prominent role of DA in WM and associative learning, PFC DA does not influence familiar associations. Blockade of D1R and D2R in the lateral PFC does not cause any behavioral deficit in monkeys remembering highly familiar stimulus-response associations (Puig and Miller, 2012, 2014; **Figures 2A,D**). This agrees with the hypothesis that DA is essential for the early stages of learning, but with extended training DA appears to play a decreasing role. So there may be a transition from goal-directed to habit-based instrumental performance likely orchestrated by the basal ganglia (Wickens et al., 2007; Graybiel, 2008).

A series of investigations carried out by the groups of AC Roberts and TW Robbins have shown in monkeys that DA depletions in another region of the PFC, the orbitofrontal cortex (OFC), disrupt conditioned reinforcement (i.e., when previously neutral stimuli in the environment become associated with reward). After DA depletions restricted to the OFC monkeys were insensitive to conditioned reinforcers and persisted responding in the absence of reward, resembling the compulsive responding of drug addicts (Walker et al., 2009). The OFC is also critical for reversal learning, the ability to switch responding to a previously non-reinforced stimulus upon learning (Robbins and Roberts, 2007; Kehagia et al., 2010). After excitotoxic lesions of the OFC monkeys were able to learn novel stimulus-reward associations, but showed marked perseverative deficits in their ability to reverse the associations (Clarke et al., 2008). Interestingly, this was sensitive to serotonin but not DA depletions (Clarke et al., 2004, 2005, 2007). In contrast, DA, but not serotonin, depletions in the caudate nucleus disrupt reversal learning, revealing striking neurochemical dissociations between the DAergic and serotonergic neuromodulatory systems in fronto-striatal circuits (Clarke et al., 2011, 2014). The role of specific DA receptors in these effects have not been explored, so this important piece of information is missing. In this regard, one study showed that systemic blockade of D2R, but not D1R, impairs reversal learning in monkeys without affecting new leaning (Lee et al., 2007). However, administration of drugs in this study was systemic, making the specific contribution of PFC D1R and D2R to the reported effects unclear.

## RODENT STUDIES

Separate populations of PFC pyramidal neurons with unique morphological and physiological properties have been identified in mice that express only D1R or D2R (Gee et al., 2012; Seong and Carter, 2012). This is similar to the well-established direct and indirect pathways in the basal ganglia, that express D1R and D2R, respectively (Albin et al., 1989; Alexander and Crutcher, 1990; Smith et al., 1998; Gerfen and Surmeier, 2011). In fact, a recent study has demonstrated that selective (optogenetic) activation of D1R-expressing neurons in the striatum (direct pathway) promotes reinforcement learning, whereas selective activation of D2R-expressing neurons (indirect pathway) induces transient punishment (Kravitz et al., 2012). However, the specific contribution of D1R- and D2R-expressing neurons in the PFC to learning has yet to be elucidated.

Early work in rats demonstrated, as in monkeys, that elevating or depleting DA in the PFC impaired spatial WM performance (Simon, 1981; Bubser and Schmidt, 1990; Murphy et al., 1996). In keeping with studies in monkeys, there is a phasic release of DA into the PFC during delayed response tasks, the magnitude of DA eﬄux being predictive of memory accuracy (Floresco and Phillips, 2001; Phillips et al., 2004). Moreover, these DA actions are mediated by D1R. Zahrt et al. (1997) reported that overstimulation of PFC D1R with a D1R agonist induced deleterious effects in spatial WM of rats performing a delayed alternation task, an effect reversed by pretreatment with a D1R antagonist. Rats were required to alternate between two arms to obtain a reward, with a delay between trials of 5–30 s. Another study using a comparable range of delays (0–16 s) found that intra-PFC infusions of a D1R agonist, but not a D2R antagonist, could disrupt or facilitate performance in a task designed to account for the contribution of attention to WM. Importantly, this work suggested that different levels of DA may be required for different cognitive processes (Chudasama and Robbins, 2004). Seamans and Floresco used a delayed response variant of the radial-arm maze task to demonstrate, also in rats, that other types of “WM” with comparatively longer delays (in the order of 30 min to several hours) are also sensitive to manipulations of PFC D1, but not D2, receptors (Seamans et al., 1998; Floresco and Phillips, 2001; Floresco and Magyar, 2006; Floresco, 2013). We note that some of these studies aimed at directly testing whether inadequate activation of PFC D1R in rodents caused the same detrimental effects on WM previously reported in monkeys, where memory delays were in the order of few seconds. Thus, and as pointed out previously (**Box 1**), it seems like studies across species have not reached a consensus in defining what “WM” is. However, altogether, these studies implicate PFC D1R in different types of “short-term” memory.

Also on par with primate studies, insufficient or excessive activation of PFC D1R impairs the performance of rats in WM tasks following an inverted-U shaped curve (Seamans et al., 1998; Mizoguchi et al., 2009; Floresco, 2013). Interestingly, this has been recently extended to a more holistic view of the role of D1R/D2R in cortico-striatal circuits. Transgenic mice with selective and reversible overexpression of D2R in the striatum exhibit poor WM abilities that correlate with exacerbated PFC D1R activation (Kellendonk et al., 2006; Li et al., 2011). In contrast with the monkey literature, though, rodent work has suggested that PFC D2R could play a role in WM. Druzin et al. (2000) reported that intra-PFC infusions of a D2R agonist disrupt performance of rats in a delayed-response task and that this D2R modulation of WM may be linear (i.e., lower/higher levels of D2R activation are associated with better/poorer performance). Thus, PFC D2R could also contribute to WM but following distinct principles of operation than D1R (i.e., linear vs. an inverted-U modulation; Williams and Castner, 2006; Floresco, 2013). So, perhaps the effects of the D2R agonist bromocriptine observed in human studies can be attributed in part to PFC D2R.

Furthermore, D4R may be key for emotional learning. In rats, activation of D4, but not D1, receptor subtypes in the medial PFC strongly potentiates the salience of emotional associative fear memories. Furthermore, individual neurons in the medial PFC actively encode emotional learning, and this depends on D4R activation (Laviolette et al., 2005). Conversely, stimulation of D1R and not D4R blocks the recall of previously learned emotionally relevant information suggesting, again, that D1R help shape memories. So, PFC D1R and D4R may play discrete roles (memory vs. learning) in the acquisition of emotional associations (Lauzon et al., 2009).

D1R and D2R exert complex modulatory actions on the activity of PFC neurons, as shown by *in vitro* recordings in PFC slices of rodents (see for an extensive review Seamans and Yang, 2004). Briefly, DA tends to enhance spiking via D1R through Na^+^, K^+^, and Ca^2+^ currents (Yang and Seamans, 1996; Gorelova and Yang, 2000), an effect also observed in PFC slices of monkeys (Henze et al., 2000; González-Burgos et al., 2002). Conversely, DA decreases spiking via D2R, possibly through modulation of glutamatergic receptors and Na^+^ conductances (Gulledge and Jaffe, 1998, 2001; Gorelova and Yang, 2000; Tseng and O’Donnell, 2004). Moreover, stimulation of PFC D2R can also induce an afterdepolarization mediated by L-type Ca^2+^ channels and NMDA receptors (Gee et al., 2012). Besides these contributions of DA to the modulation of PFC activity, several rodent studies have also provided evidence that PFC neurons shape the activity of DA neurons. For example, Takahashi et al. (2011) found that OFC inactivation impaired state-value representations in VTA DA cell activity, in particular the effect of the animals own action plan on the state value. Furthermore, Jo et al. (2013) showed that PFC inactivation increases the DA response to reward-predicting stimuli. This matches a series of computational modeling studies in which PFC becomes part of the system that determines the value of the current state and propagates this information to the DA system (e.g., Frank et al., 2001; O’Reilly and Frank, 2006; Hazy et al., 2007). Although this supports a general role of PFC in shaping DA cell activity, the specific contribution during behavior depends on the corresponding firing patterns of the PFC neurons that affect DA cells.

## BIRD STUDIES

Higher cognitive abilities evolved largely independently in birds and mammals. This parallel evolution gave rise to several crucial differences in neural organization. While avian and mammalian striatum and pallium are homolog (derived from a common ancestor), there are considerable differences in the organization of the pallium (Jarvis et al., 2005). For instance, the avian telencephalon does not have a pallial commissure comparable to the mammalian corpus callosum. The most notable difference, however, is the lack of the typical cortical lamination in the avian pallium (Jarvis et al., 2005). In other words, in spite of a shared evolutionary ancestry and a similar functionality, the avian and mammalian “cortex” look entirely different: what has evolved into layers in the mammalian brain might have evolved into different regions in the avian brain (Jarvis et al., 2013). Other organizational principles were preserved or independently evolved. For instance, a recent analysis of the avian connectome revealed a very similar network organization between birds and mammals (Shanahan et al., 2013). Using graph theory, the authors found that the telencephalon of both species has a comparable organization into modular, small-world networks with a connective core of hub nodes. The most relevant here is the “prefrontal” hub. While the avian brain has no homolog of the mammalian PFC, it has a functional analog (structure with comparable functionality) – the NCL. A detailed comparison between both structures has been provided elsewhere (Güntürkün, 2005a,b; Kirsch et al., 2008). Briefly, PFC and NCL are centers of multimodal integration that are closely connected to all secondary sensory and motor regions (Kröner and Güntürkün, 1999).

Much like the PFC, the NCL is involved in WM as revealed by lesion studies (Mogensen and Divac, 1982; Güntürkün, 1997) and single cell recordings in pigeons during Go/Nogo tasks (Diekamp et al., 2002). Recently, an elegant study demonstrated that single neurons in the NCL of crows maintain memory information in two versions of a delayed match to sample task (DMS; Veit et al., 2014), the classical paradigm of WM research in primates. The animals were trained to view a sample image and indicate this image among similar images following a short delay (1–2.3 s). Similar experiments revealed an involvement of NCL in other cognitive functions such as categorization (Kirsch et al., 2009), the integration of time-to-reward with reward amount (Kalenscher et al., 2005), and executive control over what information is maintained in WM (Rose and Colombo, 2005). Another hallmark of prefrontal function, the processing of rules that guide behavior, was recently reported in the NCL of crows (Veit and Nieder, 2013). The authors used the same paradigm that was used in the original demonstration of such processes in primate PFC, a modified DMS task (Wallis et al., 2001). They report that single neurons in the NCL represent behavioral rules that instruct the animals how to respond to subsequent stimuli, a result that mirrors the original findings in the PFC.

The NCL, as the PFC, is the prime cortical (pallial) target of dopaminergic innervation (Durstewitz et al., 1999b). As in mammals, these projections arise in VTA and SNc (Waldmann and Güntürkün, 1993; **Figure 1**). Dopaminergic projections to the avian telencephalon show two distinct anatomical features (Wynne and Güntürkün, 1995). One type, “en passant” projections, are also found in the mammalian brain. These axons travel through the telencephalon, contacting a large number of dendrites and somata of predominantly smaller target neurons. The other type, “baskets,” has not been reported in the mammalian brain. Here, individual fibers densely wrap around the somata and initial dendrites of predominantly larger cells. Interestingly, this type of innervation might be functionally comparable to the pattern of innervation in the mammalian cortex. In mammals, large pyramidal neurons lie mainly in deeper layers and are targeted by DA terminals through their proximal (in primates also distal) dendrites. The basket structures might be a way to generate a similar innervation of larger cells in the absence of cortical organization (Durstewitz et al., 1999b). Compared to the mammalian PFC, the avian NCL contains members of both DA receptor families, with a considerably lower density of D2 compared to D1 receptors (Dietl and Palacios, 1988; Durstewitz et al., 1998).

Overall, the role of DA in the avian brain is largely comparable to its role in the mammalian brain. DA is involved in motor control and learning, and in birds it also contributes to the acquisition and control of birdsong (Rieke, 1980, 1981; Güntürkün, 2005a; Fee and Goldberg, 2011). Even though birdsong is a major focus of avian research, here we will only briefly refer to this work. It has been reviewed extensively elsewhere and the main focus of the song literature is the role of DA in basal ganglia circuits (Kubikova et al., 2010; Fee and Goldberg, 2011; Simonyan et al., 2012). To our knowledge, no study has recorded avian dopaminergic neurons during learning, so there is no direct evidence for reward prediction error coding in avian DA neurons. However, several studies provide indirect evidence for temporal discounting (TD)-learning in birds. The only study that recorded from single DA neurons in the VTA of songbirds showed that DA neurons are strongly modulated by social context. The authors interpret this result in the light of “approval” – positive feedback of the females that the male subjects sang to (Yanagihara and Hessler, 2006). Later work confirmed that such social context activity is involved in modulating the singing-related activation of the song system (Hara et al., 2007). Further evidence comes from behavioral studies. Pigeons learn a simple discrimination task faster if they receive a larger reward for correct discrimination than with a smaller contingent reward. This difference in learning rate can be predicted by different reward prediction errors due to the different reward magnitudes (Rose et al., 2009b). Furthermore, injections of D1R antagonists in the striatum abolish this effect (Rose et al., 2013). Interestingly, the birds are still able to learn the discrimination but the learning rate is no longer modulated by the contingent reward magnitude. Learning shows an average rate with a slight decrease in performance on a large reward and a slight increase in performance with a small reward.

As in the mammalian PFC, DA in the avian NCL is critically involved in mechanisms of learning and memory. DA levels in the PFC of monkeys increase during WM tasks (Watanabe et al., 1997) and, consistently, microdialysis in the NCL of pigeons show an increase in DA during a DMS task with a delay (4 s) compared to the same task without a delay (Karakuyu et al., 2007). Furthermore, injections of a D1R agonist (SKF81297) into the NCL and striatum improve performance on a DMS task (Herold et al., 2008). Interestingly, these injections were only beneficial on days with low performance; if the animals performed well, agonist injections disrupted performance. These findings are in line with the mammalian literature showing that DA modulates performance following an inverted-U shaped curve, where too much or too little D1R activation is detrimental to performance. It also complements nicely the reports showing that humans with genetically lower levels of DA in PFC are less susceptible to the detrimental effects of stress on WM (see Human Studies). In addition, and again in line with the mammalian literature on WM, injections of a D1R antagonist (SCH23390) into the NCL disrupt the ability of pigeons to focus their attention over longer periods of time and to ignore distracting stimuli (Rose et al., 2010).

In a recent study, Herold et al. (2012) assessed the expression of different DA receptor types in the NCL of pigeons trained on different cognitive tasks. This approach allowed the dissociation of changes in receptor expression due to WM (using a DMS task), stimulus selection (a stimulus-response task), or general task components such as reward and response selection. It is noteworthy that the mammalian D1R family is extended in the avian brain. In addition to D1A (D1) and D1B (D5) receptors, the avian brain also contains the receptor D1D. The authors report that general task components have no influence on D1R expression in the NCL. However, WM components increase expression of D1B and stimulus-response learning increases expression of D1A and D1D receptors. None of the task components affected the expression of D2R. These results demonstrate an involvement of DA receptors in the NCL not only in WM but also in learning mechanisms (Herold et al., 2012). In line with these results, microinjections of a D1R antagonist (SCH23390) to the NCL of pigeons resulted in severe disruptions of discrimination reversal learning (Diekamp et al., 2000). This result is in contrast to the finding that DA in caudate nucleus, but not the OFC, of monkeys is required for reversal learning (see Non-Human Primate Studies; Clarke et al., 2004, 2005, 2007, 2011).

## CONCLUSION

Despite decades of intense research, we are only now starting to comprehend the specific roles of DA in several PFC-dependent learning and memory processes. A main obstacle in understanding the complex DA modulation of PFC function, both at anatomical and physiological levels, is the outstanding heterogeneity and specificity of the DA system itself. Therefore, a cross-species comparison may contribute to identify general principles of DA function in the PFC. Each model species discussed here provides its unique advantages and challenges. Certainly, one of the main goals of studying the dopaminergic system is to expand our understanding of the healthy and diseased human brain in order to develop better treatments for neurological and psychiatric disorders with abnormal DA transmission. Since this research poses many technical constraints, non-human primates offer an alternative to study complex behavior and higher cognitive functions. In contrast, rodents can be manipulated vastly with a variety of genetic/optogenetic approaches, but their cognitive abilities might not be sufficient to address higher cognitive functions of humans. Finally, studying the avian brain offers an evolutionary perspective that might help identify crucial features and constraints of the dopaminergic system. Indeed, the crucial role of DA in executive function is highlighted by the fact that the independent evolution of higher-order cognition in birds gave rise to a largely comparable DA function – even in the absence of cortical layering.

Some major findings have been consistently replicated in different species, establishing their robustness. First, elevating or depleting DA levels in PFC impair performance in WM tasks. Second, PFC DA modulates WM via D1R. The potential involvement of D2R in WM is more controversial. Third, PFC D1R modulate WM following an inverted-U shaped curve. That is, an optimal level of D1R activation is required for adequate WM performance, and this is sensitive to changes in arousal state such as fatigue or stress. Recent studies in monkeys point to interesting extensions of these findings, but still need to be confirmed in other species and in other paradigms. They showed that the inverted-U curve modulation of WM may also occur at the level of spiking in PFC neurons, and that both PFC D1R and D2R play relevant roles in associative learning but not associative memory.

Clearly, more work will be necessary to fully understand the role of different receptor subtypes present in the avian and mammalian brains in learning and memory processes. In order to succeed, and as underscored in this review, researchers working on different disciplines and with different species will need to reach a consensus in how to define different types of learning and memory processes, paying particular attention to WM-related concepts and terminology (**Box 1**). Computational modeling could provide such unified definitions and hypotheses that are testable across species.

Importantly, recent investigations conducted in rodents have highlighted the close interaction between D1 and D2 receptors present in cortico-striatal circuits. In addition, separate populations of pyramidal neurons have been identified in the rat PFC that preferentially express only D1R or D2R, similarly to the D1R-expressing direct and D2R-expressing indirect pathways of the basal ganglia. Although the specific contribution of these PFC neuron populations to learning and memory has yet to be elucidated, the use of genetic and invasive approaches in rodents is proving to be an excellent source of information. However, non-human primate models are better suited to gain deeper insights into the role of DA in more sophisticated tasks that are closer to the human cognitive repertoire. Unfortunately, genetic manipulations and invasive approaches such as optogenetics are just beginning to be developed in primates. A rapid advancement in the development of techniques applicable to humans is especially necessary, since human studies on the role DA in learning and memory have been particularly scarce. In this regard, it is now possible to measure DA release with accurate timescales by molecular fMRI (Lee et al., 2014). We hope that these emerging technical advances in primates will allow a more detailed understanding of the roles of D1R and D2R in higher-order executive function. This will be particularly important for the development of adequate drug therapies for patients with disorders that show disrupted prefrontal DA signaling such as schizophrenia, PD, and ADHD.

## Conflict of Interest Statement

The authors declare that the research was conducted in the absence of any commercial or financial relationships that could be construed as a potential conflict of interest.
